# Supplementary figures and images for: Biomechanics of subtrochanteric fracture fixation using short cephalomedullary nails: A finite element analysis
Source: PLoS One. 2021 Jul 1;16(7):e0253862. doi: 10.1371/journal.pone.0253862 (PMC8248632; doi:10.1371/journal.pone.0253862)

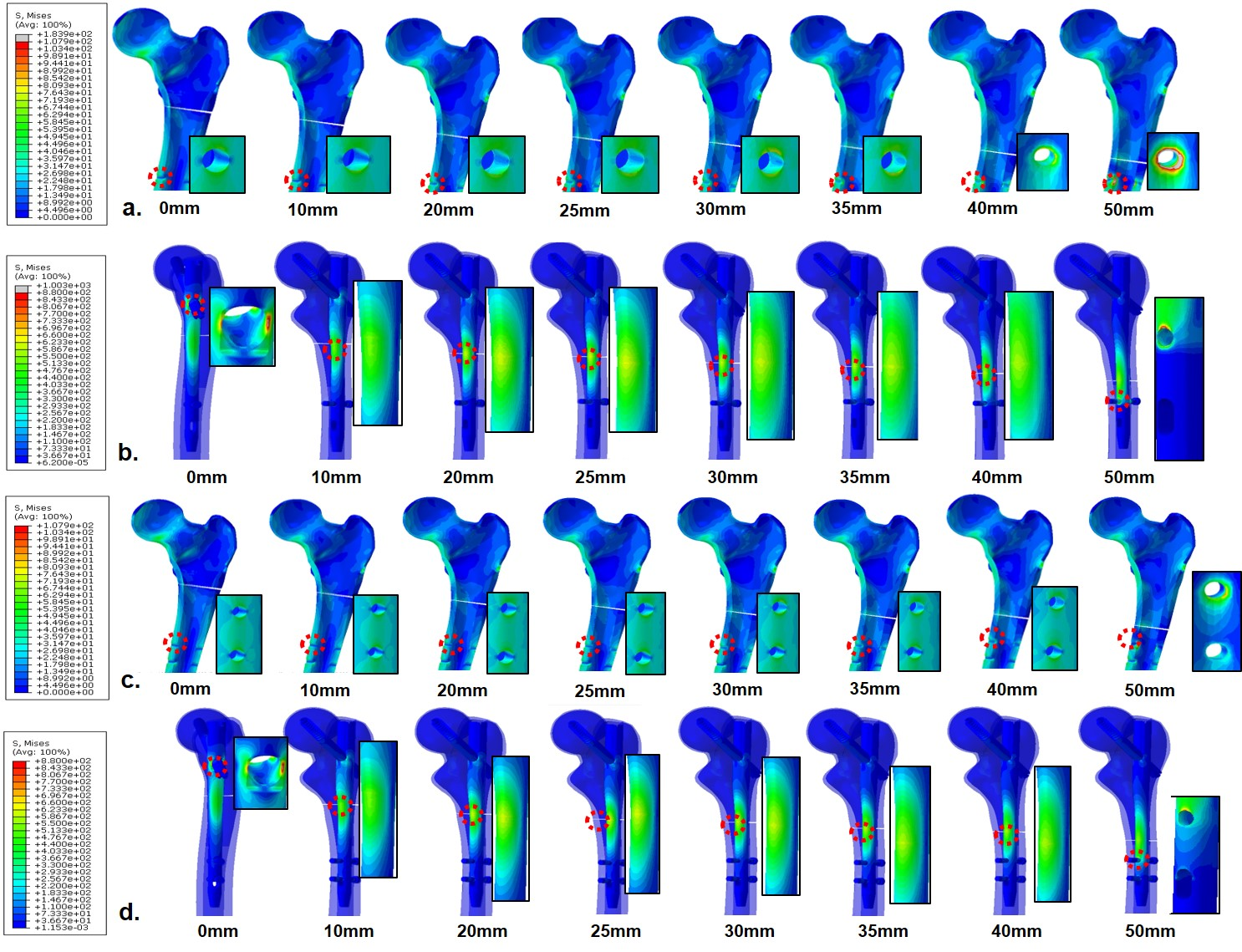

Supplement: S1 Fig — The enlarged image portion represents the point at which the peak stress was observed. (TIF) [file pone.0253862.s001.tif]

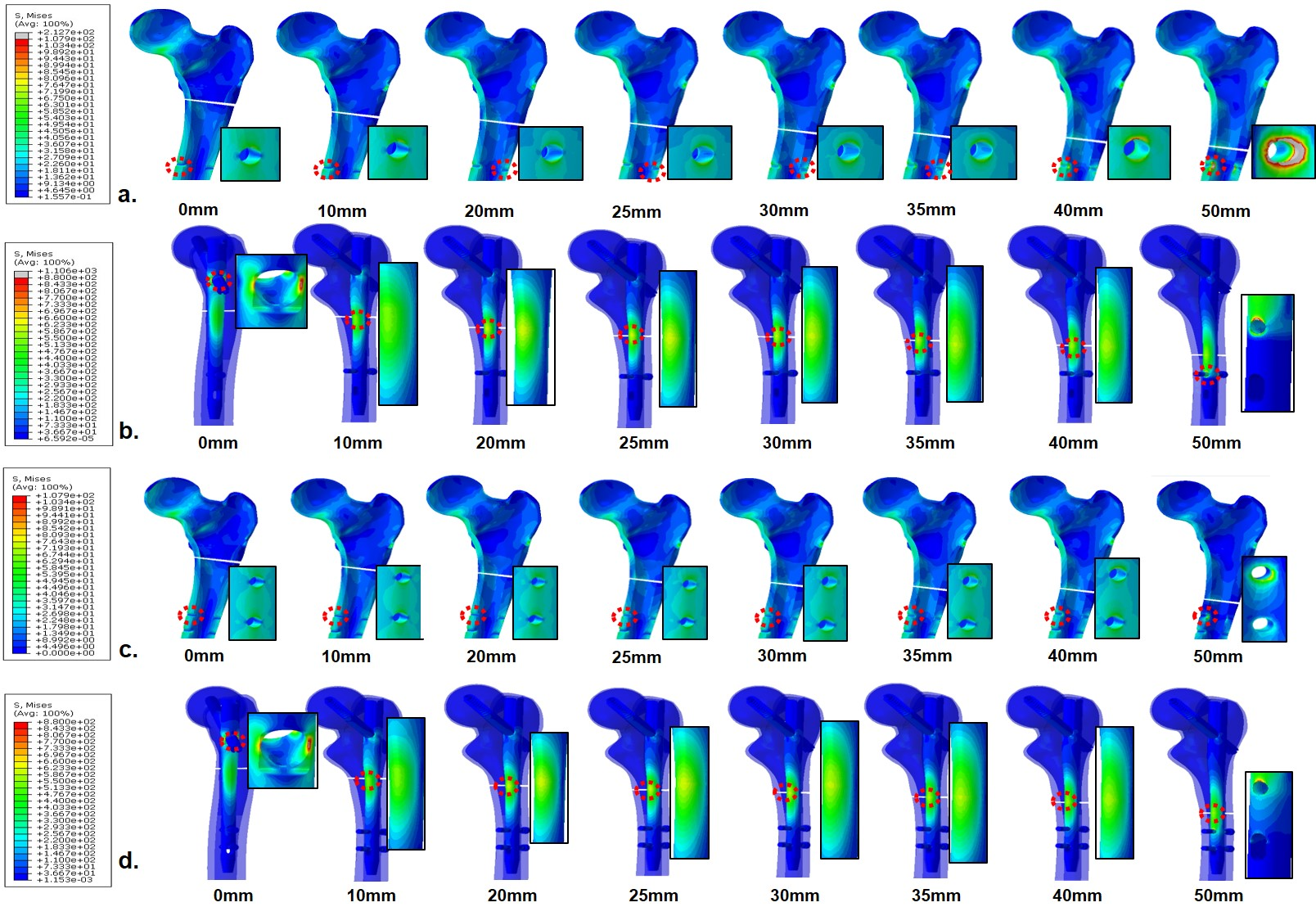

Supplement: S2 Fig — The enlarged image portion represents the point at which the peak stress was observed. (TIF) [file pone.0253862.s002.tif]

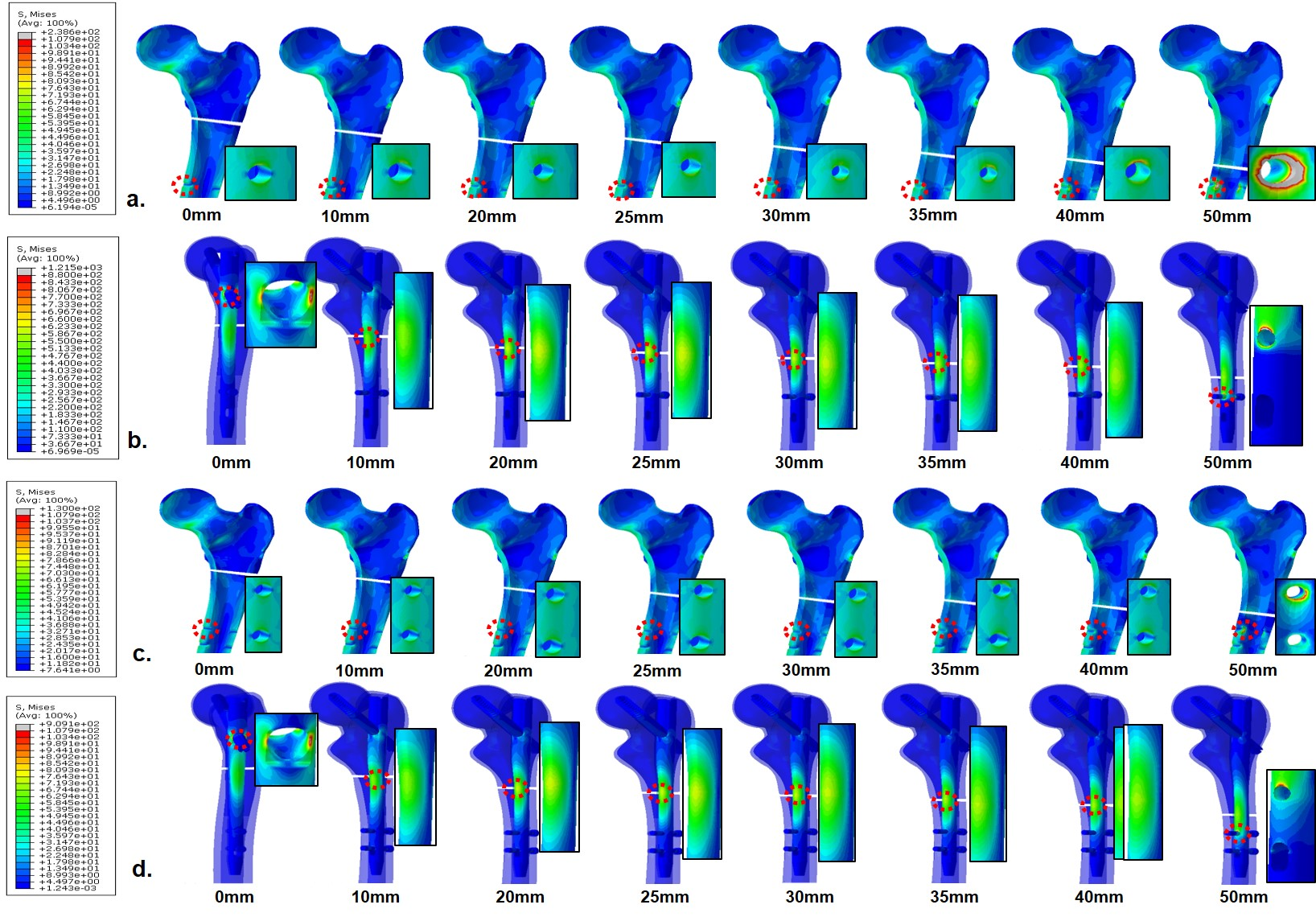

Supplement: S3 Fig — The enlarged image portion represents the point at which the peak stress was observed. (TIF) [file pone.0253862.s003.tif]
